# Screen-Printed Textile-Based Electrochemical Biosensor for Noninvasive Monitoring of Glucose in Sweat

**DOI:** 10.3390/bios13070684

**Published:** 2023-06-27

**Authors:** Safoora Khosravi, Saeid Soltanian, Amir Servati, Ali Khademhosseini, Yangzhi Zhu, Peyman Servati

**Affiliations:** 1 Flexible Electronics and Energy Lab (FEEL), Department of Electrical and Computer Engineering, University of British Columbia, Vancouver, BC V6T 1Z4, Canada; safoorak@ece.ubc.ca (S.K.); saeid.soltanian@ubc.ca (S.S.); 2 Terasaki Institute for Biomedical Innovation, Los Angeles, CA 90064, USA; khademh@terasaki.org; 3 Materials Engineering Department, University of British Columbia, Vancouver, BC V6T 1Z4, Canada; aservati@texavie.com

**Keywords:** wearable electronics, textile-based sensor, noninvasive glucose detection, electrochemical sensors, screen-printing

## Abstract

Wearable sweat biosensors for noninvasive monitoring of health parameters have attracted significant attention. Having these biosensors embedded in textile substrates can provide a convenient experience due to their soft and flexible nature that conforms to the skin, creating good contact for long-term use. These biosensors can be easily integrated with everyday clothing by using textile fabrication processes to enhance affordable and scalable manufacturing. Herein, a flexible electrochemical glucose sensor that can be screen-printed onto a textile substrate has been demonstrated. The screen-printed textile-based glucose biosensor achieved a linear response in the range of 20–1000 µM of glucose concentration and high sensitivity (18.41 µA mM^−1^ cm^−2^, R^2^ = 0.996). In addition, the biosensors show high selectivity toward glucose among other interfering analytes and excellent stability over 30 days of storage. The developed textile-based biosensor can serve as a platform for monitoring bio analytes in sweat, and it is expected to impact the next generation of wearable devices.

## 1. Introduction

Wearable devices have attracted considerable attention because of their ability to provide valuable information about the health and performance of individuals outside of clinics [1,2,3,4,5,6]. Among all wearable devices, glucose sensors are of special importance as diabetes is one of the most prevalent chronic diseases that affect the lives of many patients. Diabetes is among the top ten causes of death in the world [7]. Moreover, uncontrolled diabetes can lead to many serious complications, including coronary artery disease, nephropathy, and retinopathy [8,9]. To avoid these complications, patients are required to take medications regularly based on the quantitative measurement of blood sugar [10]. Hence, regular glucose measurements could be an essential part of the treatment and daily lives of diabetic patients. However, conventional methods of measuring glucose levels are still invasive and require skin piercing for a blood draw, which can be painful and inconvenient and can cause infection. Hence, it is important to develop a noninvasive system that is easy to use and safe for these patients [11,12].

One important proposal is to quantify the glucose level of the sweat on the skin surface. Sweat is the most readily available body fluid that contains biologically relevant ions, small proteins, and small molecules that provide valuable information about health status, such as glucose [13,14,15,16]. Several reports show a consistent correlation between sweat and blood glucose levels [17,18,19]. In addition, sweat glucose sensors offer an easy and noninvasive method [20]. Hence, sweat can be used as an alternative body fluid for noninvasive glucose detection.

Developing soft and comfortable sensors for real-time monitoring of glucose levels in sweat is essential. A growing number of research efforts have been directed toward developing innovative wearable glucose monitoring platforms, such as patch-based or textile-based designs [12]. The most popular configuration is patch-based wearable sensors, which have easy assembly and operation [21,22]. Textile materials are considered a promising new version of wearable electronics as they offer a number of attractive features, such as lightweight, breathable, comfortable, stretchable, low cost, eco-friendliness, conformal contact for interfacing with the human body, and fatigue resistance [23,24,25,26,27].

The current state-of-the-art textile-based electrochemical sensors use conductive polymers and conductive silk yarns, which are woven into fabrics [28,29]. In 2016, Liu et al. developed an embroidered electrochemical sensor integrated into a t-shirt using knitting and weaving technologies. The sensor featured flexible and mechanically robust electrodes for detecting glucose and lactate in buffer and whole blood samples [30]. A new way of fabricating glucose sensors is to use gold fibers for high conductivity and stretchability [31,32]. Nevertheless, there is a trade-off between softness and the robustness of the yarn and fiber-based sensors. Even though some improvements have been made regarding the robustness of these sensors, they are still limited to simple electrode geometry and substrates, and neither type is commercially available [33].

The screen-printed sensors, on the other hand, provide greater robustness as well as the ability to design more complex sensor shapes. Screen printing is a scalable, fast, and efficient way of manufacturing microelectrodes at a low cost. Regarding the materials, with the development of a wide variety of functional inks, the application of the screen-printing technique for electrochemical sensors has witnessed great progress. Nanoparticle-based inks of many conductive materials have been developed, such as Ag [34], Au [35], and Cu [36], for printed electronics. These materials have been found to be highly effective in producing stable and high-performance electrochemical sensors. Moreover, since this technique already exists for printing clothing, no major investment or research and development costs are required for its incorporation into clothing manufacturing for mass production [37,38]. This makes the technology highly accessible and attractive for industrial applications.

In recent decades, different types of noninvasive glucose sensors based on sweat have been developed, such as colorimetric [39,40], optical [41], electrical [42], and electrochemical sensors [43]. However, despite their potential benefits, these sensors have some limitations that have hindered their widespread adoption. The principles underlying these technologies are often complex, making their designs difficult and relatively expensive. In contrast, electrochemical sensors are widely used due to their reliability, ease of use, and quantitative measurement capabilities with a fast response time [44].

It is essential to have a sensor that is sensitive only to glucose levels among the myriad analytes in sweat. As a result, selectivity is an important factor to consider in designing the sensor. Enzyme-based sensors are considered to be the best candidates for high selectivity and sensitivity in glucose monitoring [45,46]. The most commonly used enzyme in glucose biosensors is glucose oxidase (GOx), which exhibits high selectivity towards glucose as well as the ability to withstand a variety of pH and temperature conditions.

Herein, we demonstrate a fully integrated, enzymatic electrochemical sensor for sweat glucose detection on a fabric substrate. The fabrication process of electrodes is low-cost screen-printing for highly flexible textiles and compatible with mass manufacturing in comparison to existing textile-based sensors. The sensor consists of three screen-printed electrodes with carbon and silver paste. The fabric-based sensor offers an inexpensive and customizable platform that can be used for single-use and continuous biomarker measurements for individual health monitoring. We investigate the performance of the sensors and demonstrate high sensitivity and selectivity toward glucose in emulating human sweat. We demonstrate the high durability and robustness of the sensor, which highlights the potential for these sensor designs and manufacturing for direct integration into daily clothing for monitoring glucose and other analytes.

## 2. Materials and Methods

### 2.1. Reagents and Apparatus

Chitosan, bovine serum albumin (BSA), D- (+) glucose, glucose oxidase from Aspergillus niger, Nafion perfluorinated resin solution, L-ascorbic acid (AA), uric acid (UA), lactic acid (LA), and acetaminophen (AP) were obtained from Sigma–Aldrich. Potassium chloride, potassium phosphate dibasic, and potassium Phosphate monobasic were purchased from Fisherbrand. Silver/silver chloride (Ag/AgCl) paste was obtained from ERCON INC. E2414. Carbon-graphite mediator paste (C2070424P2) was purchased from Gwent Group, UK. Dragon Skin^TM^ silicone rubber compound (DS) was used for the confinement of electrodes. All reagents were of analytical grade and were used without further purification.

### 2.2. Fabrication of Working Electrode

The electrodes were fabricated by a screen-printing method on a breathable 88%, 12% spandex fabric. Using AutoCAD, three stencils were designed, then laser cut on PET sheets with a thickness of 100 µm.

**Figure 1:**
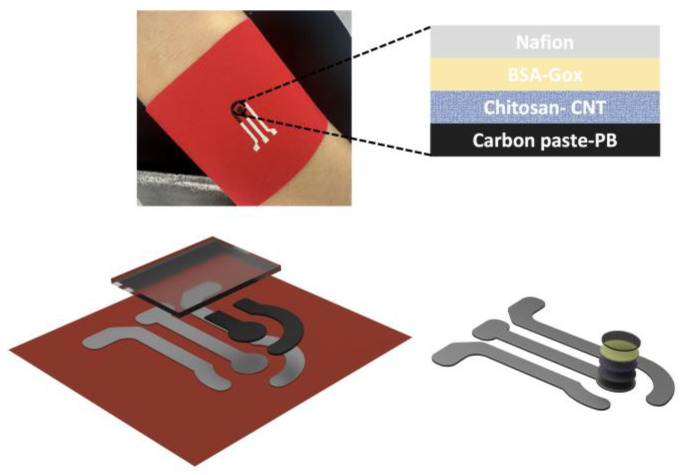
Schematic representation of textile-based glucose sensor. The optical image of the screen-printed electrodes on fabric. The system consists of silver/silver chloride reference (Ag/AgCl), Prussian blue carbon paste (PB-carbon paste) counter, and working electrodes. A transparent insulating layer confines the surface of active parts of the electrode. The schematic illustration of the modified working electrode with chitosan, Gox, and Nafion.

**Figure 2:**
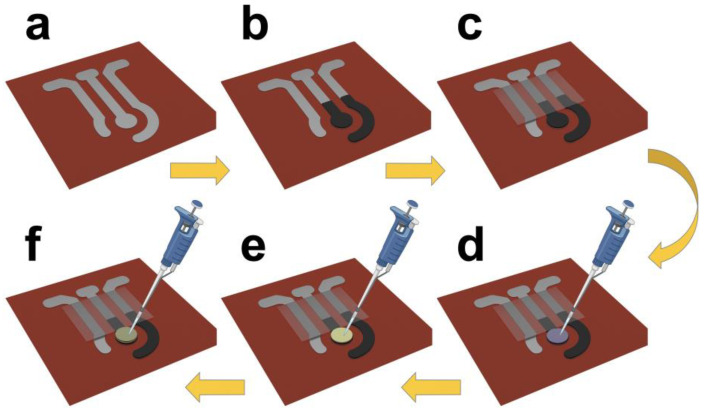
Schematic of the fabrication process of the textile-based glucose sensor. (**a**) Screen-printing of patterned Ag/AgCl as reference electrode and conductive wires for working and counter electrode. (**b**) Screen-printing of patterned PB-carbon paste as working and counter electrodes. (**c**) Confining the electrodes with a transparent isolating layer. (**d**) Drop-casting chitosan and CNT onto the working electrode. (**e**) Drop-casting GOx for functionalization. (**f**) Drop-casting Nafion as an encapsulation layer.

The sensors were printed with customized stencil masks. To begin, the Ag/AgCl conductive paste was screen printed on the fabric and baked in an oven for 30 min at 60 °C to cure the conductive ink. In the next step, the Prussian blue carbon paste (PB-carbon paste) working electrode and counter electrode were printed and cured in an oven for 30 min at 60 °C. To confine the electrode pattern and contact areas, a layer of DS was screen-printed on top of the electrode pattern. The DS layer was then cured at ambient temperature for 30 min. Figure 1 shows the flexibility and conformability of the screen-printed textile-based biosensor to the skin as well as the layered structure of the sensor. To further avoid the possible false errors made by displacement and/or deformation, such textile sensors can be worn on the body in the form of skin-tight garments, wristbands, or headbands. Additionally, stretchable fabrics, including single or double jersey or nylon spandex fabrics, can be employed to improve the stretchability of the current textile biosensing platform, enabling high stability and reliability during the mechanical deformation process. A schematic representation of the fabrication process of the electrodes on a textile substrate is shown in Figure 2.

### 2.3. GOx Immobilization

In order to prepare 1 wt.% of chitosan solution, chitosan was dissolved in 50 mM acetic acid with magnetic stirring for one hour. Then, the chitosan solution was mixed with single-walled carbon nanotubes for 30 min. The working electrode was first drop-cast with 2 µL of chitosan solution. The electrode was then functionalized with GOx solution, composed of 2 µL of glucose oxidase solution (50 mg/mL) and bovine serum albumin (15 mg/mL), and dried in ambient conditions for 30 min. Finally, 2 µL of 0.5 wt.% Nafion was drop-cast onto the electrodes, and the sensors were then allowed to dry overnight at 4 °C.

### 2.4. Characterization

A potentiostat VMP-300 (BioLogic, Seyssinet-Parisset, France) was used for all electrochemical measurements. A cyclic voltammetry (CV) test with a scan rate of (10–50 mV S^−1^) was performed on Prussian blue carbon paste (PB-carbon paste) electrodes in 0.1 M potassium chloride solution to determine CV curves. To evaluate the successful immobilization of GOx, PB-carbon paste electrodes with chitosan, BSA, and Nafion drop-casts (no GOx was used in electrode functionalization) were compared with the fabricated glucose sensors. The electrodes were tested in a 0.1 M PBS solution (pH 7.4) with glucose concentrations ranging from 0 mM to 1 mM.

Typically, sweat contains glucose concentrations between 0 and 1000 µM [47,48]. Thus, the performance of the glucose sensor was monitored in this range of glucose concentration in a 0.1 M PBS solution (pH 7.4). The amperometric response was evaluated for the glucose sensor at a low potential of −0.1 V. The performance of the glucose sensor was also evaluated by the sequential measurement of glucose concentrations at increasing levels of concentration. Starting at a 0 mM glucose concentration, the concentration was gradually increased to 1 mM step by step. Between each step after the addition of glucose, there was a 30 s pause to mix the solution.

The selectivity of the sensor was evaluated in PBS in the presence of interfering species such as ascorbic acid, lactic acid, acetaminophen, and uric acid in the solution. We evaluated the stability of the sensor’s response to repeated glucose measurements. A test of the storage stability of sensors was also performed over 30 days using six sets of three sensor batches. When not in use, sensors were stored at 4 °C. Finally, the flexibility of the sensor was tested over multiple cycles of bending with a bending radius of 8 mm.

### 2.5. On-Body Validation of Textile-Based Glucose Sensor

Epidermal evaluation of the textile-based sensor was performed on a healthy individual with no diabetes or pre-diabetic condition. The textile-based sensor was mounted on the arm of the subject for all on-body validations. The volunteer was instructed not to consume any sugar for 4 h prior to the tests. The subject underwent a light exercise of 30 min of cycling at a constant intensity. The glucose level was measured when the generated sweat was sufficient for the measurement. To verify the performance of the textile-based sweat glucose sensor, the subject’s blood glucose was measured and recorded through a commercial glucometer (Caretouch, Brooklyn, NY, USA) before every set of measurements of the textile-based glucose sensor. Then, the individual consumed a high-sugar beverage and meal, rested for 30 min, and then underwent another 30 min of cycling, and the sweat glucose was measured at the completion of the exercise.

## 3. Results and Discussion

### 3.1. System Design of Wearable Textile-Based Glucose Sensor

The design of the textile-based glucose sensor is shown in Figure 1. The sensor consists of a reference electrode made of Ag/AgCl and counter and working electrodes made of PB-carbon paste. The working electrode is functionalized with chitosan-SWCNT, BSA, Gox, and Nafion. Nafion provides a barrier against interference from other analytes to enhance the selectivity of the sensor. The fabrication process of the ultra-flexible sensors on textile substrates is illustrated. The fabrication method is an easy and low-cost method for scalable manufacturing, and the textile substrate allows for ultra-flexibility, breathability, and comfortability.

### 3.2. CV Characterization of PB-Carbon Paste

**Figure 3:**
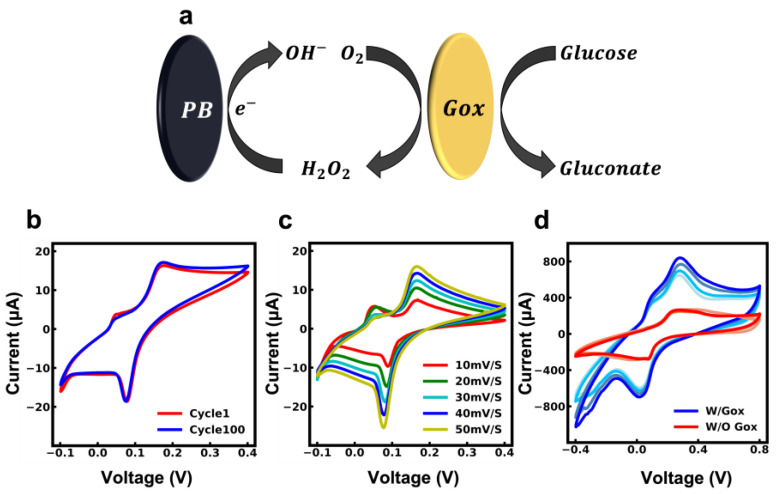
(**a**) Schematic illustration of amperometric detection mechanism of glucose on the working electrode. (**b**) CV of PB-carbon electrode for stability evaluation over 100 cycles in 0.1 M KCl solution. (**c**) CV curves of PB-carbon electrodes at various scan rates (10–50 mV/s). (**d**) Comparison of PB-carbon electrode with the fabricated glucose sensor for GOx immobilization evaluation in PBS with different glucose concentrations.

Equations (1) and (2) illustrate the working mechanisms of Gox-based sensors. First, the glucose oxidizes in the presence of Gox and produces hydrogen peroxide and gluconic acid. Then, hydrogen peroxide is reduced over the surface of the electrode, producing a current that is proportional to the glucose concentration. In order to facilitate the reduction of hydrogen peroxide at low potential, a mediator is required [11]. PB is known for its high selectivity and catalytic activity in the reduction of hydrogen peroxide (H_2_O_2_), which results in a change in the cathodic current [49,50]. The hydrogen peroxide can be detected in the presence of PB at an applied potential of around 0.0 V, which prevents electrochemical interference from other analytes present in sweat. As a result, PB-carbon paste was used as a working electrode to reduce the potential and thereby enhance the selectivity and sensitivity of the sensor. Figure 3a illustrates the process of detecting glucose using the PB-carbon electrode.

(1) $$Glucose+H_{2}O+O_{2}\overset{GOx}{\to }Gluconic\;Acid+H_{2}O_{2}$$

(2) $$H_{2}O_{2}\overset{Prussian\;blue}{\to }H_{2}+O_{2}+2e^{-}$$

To evaluate the performance of PB in the carbon electrode, the CV curve of the working electrode over 100 cycles was assessed in a 0.1 M KCL solution with a scan rate of 50 mV S^−1^ in the range of −0.1 to 0.4 V. The CV in Figure 3b shows a pair of cathodic and anodic peaks that correspond to the reversible conversion of PB to Prussian white [50,51]. Over 100 cycles, the peak currents of the PB-carbon electrode were found to maintain a similar value, indicating that the PB in the carbon paste is stable. Figure 3c illustrates the CV curve of the PB-carbon electrode at various scan rates (10–50 mV S^−1^). The results indicate that the peak of currents linearly increases with the square root of the scan rate, which is typical for a quasi-reversible redox process, showing this reaction is a diffusion-controlled process, and the electrode has good electrocatalytic activity towards the reduction of hydrogen peroxide [52,53].

### 3.3. CV Characterization of the Glucose Sensor in the Presence and Absence of GOx

After the characterization of the PB-carbon paste electrode, the glucose sensor was fabricated by drop-casting the GOx solution according to the instructions in the previous section. In order to evaluate the successful immobilization of GOx, the fabricated glucose sensor was compared with the PB-carbon electrode with chitosan and a BSA drop cast on it (No GOx was used in electrode functionalization). The CV experiment was carried out in a 0.1 M PBS solution (PH 7.4) with glucose concentrations in the range of 0 mM to 1 mM. Figure 3d shows the CV response of the glucose sensor and non-functionalized PB carbon electrode. In glucose sensors with GOx present, a significant difference in the redox and oxidation peaks and a remarkably higher current in the CV curve are evidence of the effective immobilization of GOx.

### 3.4. Electrochemical Characterization of Textile-Based Glucose Sensor

**Figure 4:**
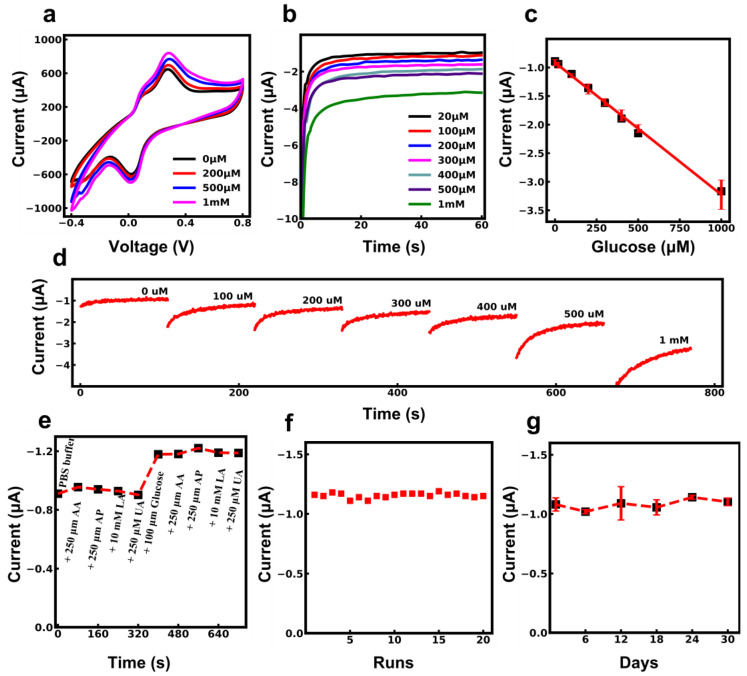
Electrochemical characterization of the glucose sensor. (**a**) CV response of glucose sensor in PBS solution at different concentrations of 0 to 1000 µM of glucose. (**b**) Chronoamperometric response of glucose sensor at −0.1 V in the solution of 0.1 M PBS with glucose concentration varying from 0 µM to 1000 µM. (**c**) Calibration plot of the amperometry response. (**d**) Amperometry response of sensor for the sequential addition of glucose to 0.1 M PBS. (**e**) Selectivity of the sensor toward the addition of interfering substances. (**f**) Reproducibility of the sensor over 20 repetitive amperometric measurements at 100 µM concentration of glucose. (**g**) Storage stability of the sensor over 30 days.

The electrochemical characterization of glucose sensors was measured in the biological range corresponding to glucose in human sweat. The CV response of the glucose sensor in PBS solution is shown in Figure 4a. The anodic current increases as glucose concentration increases from 0 to 1000 µM, indicating a glucose reaction with GOx and PB catalytic activity.

The chronoamperometric response of the glucose sensor at −0.1 V for 60 s on the working electrode in a solution of 0.1 M PBS with glucose concentrations varying from 0 to 1000 µM was tested. Figure 4b demonstrates a high sensitivity for glucose as the current proportionally increases with concentrations of glucose, displaying a remarkable sensing limit of up to 20 µM. The resulting calibration plot in Figure 4c shows a linear response toward glucose in the desired range with a sensitivity of 18.41 µA mM^−1^ cm^−2^ (R^2^ = 0.996). The sensitivity of the sensor was calculated by referring to Equation (3) and dividing the slope of the calibration curve by the surface area of the electrode. The slope of the calibration curve was calculated as 2.276 μA/mM, and the radius of the working electrode was 2 mm. The error bars represent the standard deviations of the measured data for three samples. Moreover, only a small amount of sweat (approximately 10 μL) is needed to initiate the functionality of our textile biosensing platform, facilitating its practical measurement. In addition, to maintain sensing accuracy when the body sweats less, immediate placement of pilocarpine delivery gel with the device will be applied to the conditional area to ensure sufficient and stable sweat for continuous sensing [3].

(3) S=Slope/Active surface area

Further evaluation of the glucose sensor’s performance was done using consecutive measurements with increasing glucose concentration to simulate on-body functional operation. In Figure 4d, the amperometry response of the sensor is shown after successive additions of glucose to PBS. The concentration of glucose in the solution was increased by 100 µM in each step, starting from 0. Between each step, there was a 30 s pause for glucose addition and mixing. The resulting curve shows that the current increases with the glucose addition in each step.

The proposed textile-based sensor is designed to measure glucose levels in sweat. As a result, it is crucial that the sensor be highly selective for glucose. The common interfering analytes in human sweat are AA, LA, AP, and UA. Therefore, these analytes, in their respective physiological concentrations, were spiked onto the sensors. The measurement started in PBS buffer solution, and different interfering analytes in the concentrations of 250 µM of AA, 250 µM of UA, 250 µM of AP, and 10 mM of LA were added to the solution. Data recordings were paused for 30 s for each analyte addition.

Figure 4e and Supplementary Figure S1 show that interfering analytes caused minor fluctuations in the amperometry response compared to glucose addition, which shows negligible effects of interfering analytes on the electrochemical response of the sensor. This high specificity toward glucose proves the efficiency of the PB mediator, the low working potential of −0.1, and the Nafion coating that eliminates potential interferences [54,55].

### 3.5. Stability Evaluation

The stability of the sensor is another essential factor for wearable glucose sensors. Storage and operational stability tests were performed on the sensors. The operational stability of the biosensor was tested over repetitive measurements at a 100 µM concentration of glucose. Figure 4f shows that the sensor’s response to multiple glucose measurements remained the same, with a 3.8% relative current change between repetitive measurements.

Aside from operational stability, the storage stability of the sensor was evaluated by keeping the fabricated sensor at 4 °C in a fridge. Six sets of three sensors were fabricated. Every six days, one set was tested in a solution of 100 µM glucose. The long-term stability results in Figure 4g indicate that the sensor’s performance is consistent over a period of 30 days of storage at 4 °C. The formula to calculate the relative current change is shown below.

(4) $$Relative\;current\;change=\frac{I-I_{0}}{I_{0}}$$

### 3.6. Flexibility Evaluation

**Figure 5:**
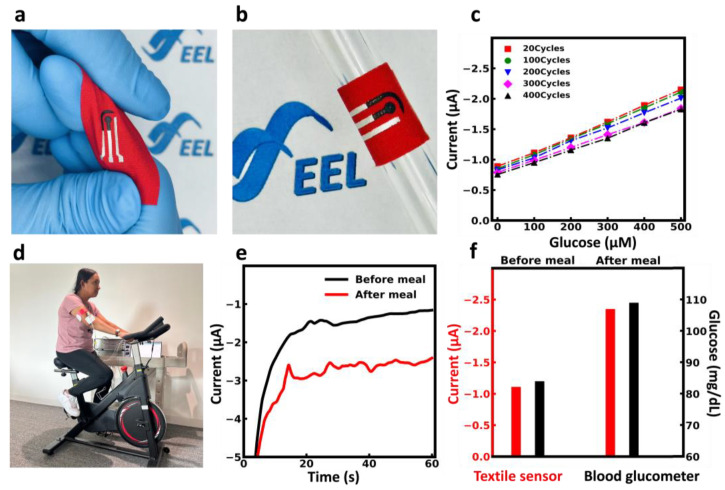
Flexibility test of the wearable glucose sensor. (**a**) Photograph of the textile-based sensor under mechanical twist. (**b**) Photograph of the flexible textile-based sensor in a bent state on a metal rod (radius of curvature = 4 mm), (**c**) Calibration graph of glucose sensor under different cycles of bending. (**d**) Photograph of the subject wearing a textile sensor during stationary cycling. (**e**) Electrochemical signal recording of glucose levels before and after sugar intake in the body. (**f**) Comparison of glucose levels in human sweat using a textile-based glucose sensor (red) and in blood using a commercial blood glucometer (black). Photo credit: Safoora Khosravi.

Flexibility is a critical factor for real-life on-body applications where the sensor is subjected to mechanical deformation. In order to evaluate the flexibility of the sensor under mechanical deformation, the sensors underwent mechanical twisting and bending (Figure 5a,b). The performance of the sensor for 400 cycles of bending at a bending radius of 4 mm was evaluated (Figure 5c). The sensor response was recorded after 20, 100, 200, 300, and 400 cycles of bending in glucose concentrations of 0–500 µM. The amperometric results of the sensor show that the sensor can withstand over 200 cycles of bending without significant changes in its performance. After 400 cycles of bending, the relative current change was less than 15%, demonstrating excellent sensor stability over mechanical deformation. This is attributed to the high flexibility of the substrate and the good adhesion of the carbon and Ag/AgCl paste to the substrate.

### 3.7. On-Body Measurement

To validate the performance of the textile-based glucose sensor, we monitored the fluctuation in sweat glucose levels both before and after a meal. To illustrate the immediate impact of glucose intake on human perspiration, the human subject consumed foods and beverages with high sugar content, and subsequently, the changes in sweat glucose levels pre- and post-sugar consumption were monitored.

Figure 5d and Supplementary Figure S2 shows a human subject wearing the textile sensor on the armband. To study this effect, the subject did not consume any sugar for 4 h prior to the test. The monitoring was performed 30 min after a light exercise of cycling with the textile-based glucose sensor. The accuracy of on-body measurement was evaluated by comparison of the textile-sensor readings with those of the commercial glucometer. The subject then consumed a high-sugar meal and beverage and, after 30 min of rest, started to cycle for 30 min. The sweat and blood glucose levels were monitored with a textile-based glucose sensor and glucometer, respectively. Figure 5e demonstrates the amperometry response of the sensor before and after the meal, which shows an increase in the amperometry response of the sensor.

Figure 5f shows the comparison between the glucose level in sweat using the textile-based glucose sensor and in blood using a commercial glucometer. The responses of both sensors demonstrated a steep spike in both sweat and blood glucose levels. Therefore, we can conclude that the textile sensor can reliably monitor changes in human glucose levels, and with accurate calibration, the sensor holds significant potential for measuring sweat glucose levels.

## 4. Current Challenges and Future Perspectives for the Wearable Textile Biosensing Platform

Despite the recent advances in wearable textile biosensors, there are several issues to be evaluated before a sweat biosensor can be commercialized. For example, sweating is a complex process that involves fluctuations in pH and temperature during physical activities (exercises), in local sweat extractions, or even varying significantly between individuals (approximately two units for pH and about 20–40 °C for temperature [16,56,57]. Such variations may impact the biosensor’s performance, particularly by impeding the activity of the enzyme that the sensor relies on. Hence, evaluating these effects comprehensively in off-site, off-body, and on-body environments is essential to ensuring reliable and accurate biosensing performance. We have summarized the recent advances that have been explored to address changes, which may provide some guidance for researchers who are working in the field of wearable biosensors.

Environmental fluctuations of temperature and pH: A reliable measurement in sweat biosensors, especially those based on an enzymatic element whose activity strongly depends on environmental parameters, including pH and temperature. To maintain high accuracy that is independent of those environmental changes, dynamic correction of pH, temperature, and sweat rate is needed. For example, real-time correction using in situ pH measurements provides more accurate glucose monitoring data than those without corrections compared with the real glucose concentration measured by the glucose assay [58]. Similarly, wearable glucose analysis in sweat with the incorporated pH and temperature correction according to local dynamic fluctuations in sweat during on-body tests realizes a highly accurate measurement [56]. In addition, the combination of real-time corrections based on pH, temperature, and humidity measurements is reported to further maximize the accuracy of the biosensors.

Insufficient sweat rate: another challenge is to obtain substantial volumes of sweat from sedentary and elderly individuals for on-demand and in situ analysis or in cold environments, which impedes the use of sweat biosensors in such real-life scenarios where they have limited access to sufficient sweat. Iontophoresis has been employed to enhance sweat production. Such iontophoresis involves the delivery of stimulating agonists to the sweat glands with the aid of an electrical current. Moreover, such a method can be programmed to control sweat generation at different secretion rates at a selected site [3,59]. In addition, sweat-stimulating drugs (i.e., pilocarpine), along with iontophoresis, are also reported to consistently improve the secretory rate [13].

Skin surface contamination, old and new sweat mixing problems: The skin may be contaminated by dead skin cells, sebum, the condensate from trans-epidermal water loss, and other contaminants induced by human activities or environmental exposure. The combination of flexible microfluidics with well-sealed microchannels or microchambers has been applied to harvest sweat to decrease contamination and evaporation of sweat samples (similar to wearable tear analysis) [60,61,62]. In addition, patterned hydrophilic fillers were designed to realize rapid sweat uptake at low secretion rates. In addition, some work reports that the sensor is in close and direct contact with the skin and sweat glands, facilitating the reduced measurement time.

Mixing and carry-over between new and old sweat samples were also factors affecting sensor accuracy. Without a system for continuous sweat flow to refresh the sensing sample and control sample evaporation and volume, the biosensor may have less reliable continuous readings. Pressure-driven microfluidics can empower a consistent sweat flow, and fresh microliter sampling volumes can be guided to the sensor surface to minimize old and new sweat mixing issues [63]. Therefore, a microfluidic-integrated wearable biosensing platform has been emerging to achieve much more reliable wearable analyte monitoring with excellent practicability [64,65].

Hypoglycemia and hyperglycemia: The finger-pricking approach, one of the most widely used self-monitoring methods, is enzymatic-based and involves sampling blood from a finger, which is not a continuous measurement and requires multiple intervals throughout the day to manage the glucose levels. Such a non-continuous method may overlook periods of hyperglycemia and hypoglycemia, which occur outside of the sampling window. Noninvasive, continuous sweat analyte monitoring based on a wearable biosensor platform can generate a large amount of data that includes clinical information indicating hyperglycemia or hypoglycemia. Modern and powerful computational, information, and communication technologies, particularly advanced machine learning algorithms, could be used to manage such large volumes of data resulting from high-frequency measurements. Such novel data analysis and machine learning algorithms include support vector machines to distinguish diabetes from healthy controls [66,67], neural networks to diagnose the severity of diabetes [68,69], or K-means to differentiate between different subtypes of diabetes, including hypoglycemia and hyperglycemia [70,71].

**Figure 6:**
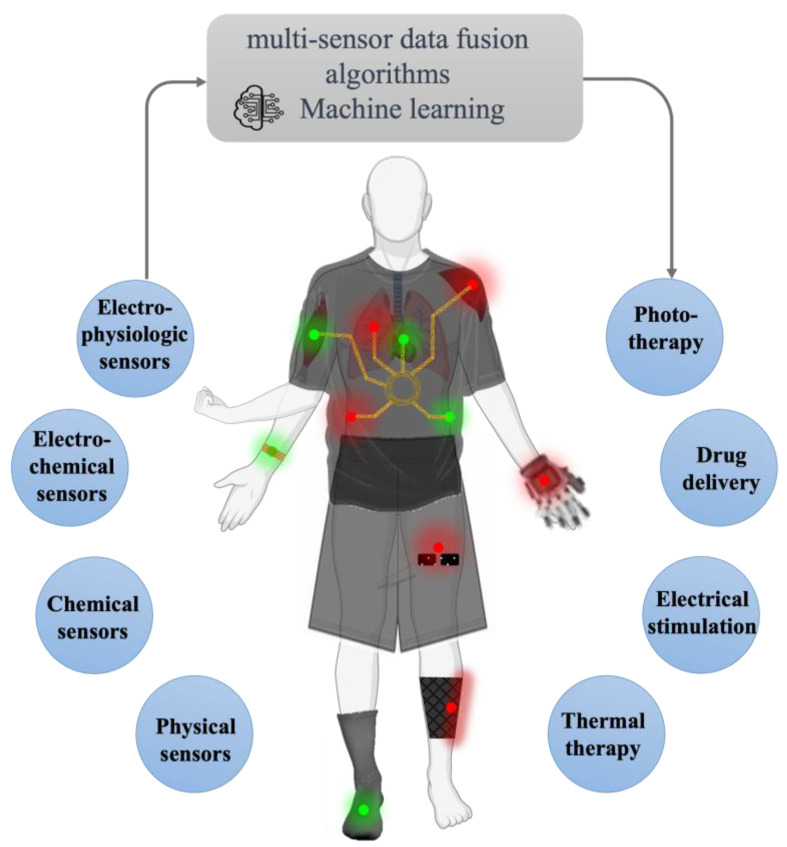
Vision of a future closed-loop wearable textile platform for diagnostic and therapeutic applications.

Overall, compared to other sweat-sensing modalities [24], textile-based wearable platforms offer natural breathability, facilitating the process of sweating and evaporation to cool the body. Moreover, they possess remarkable flexibility, softness, and comfort. Such a textile-based biosensing strategy can be integrated into existing garments, including socks, pants, and gloves, enhancing the versatility of sweat monitoring with excellent practicability. As shown in Figure 6, our vision entails a futuristic textile-based wearable platform that integrates continuous chemical monitoring of biomarkers of interest from sweat along with physical and physiological monitoring of vital signs. This advanced platform would incorporate multi-sensor data fusion algorithms to process the collected information. Notably, it would achieve an autonomous and feedback-controlled “sense-to-act” capability. Therefore, this on-demand therapeutic delivery would be modulated based on the comprehensive data obtained from the sensor network. Such an integrated platform holds great potential for revolutionizing healthcare through personalized monitoring and treatment approaches.

## 5. Conclusions

We demonstrated a screen-printed electrochemical sensor on a textile substrate for monitoring glucose levels in sweat. The fabrication method is cost-effective and can be easily integrated with everyday clothing using existing clothing manufacturing processes at an industrial scale. The sensors are fabricated layer-by-layer with a screen-printing method that enables the fabrication of customized electrode geometries and configurations. The biosensor shows excellent detection performance toward glucose with a high sensitivity of 18.41 µA mM^−1^ cm^−2^ in a linear range over 0–1000 µM (R^2^ = 0.996). Moreover, the sensor showed a high selectivity for glucose without interference from other analytes present in human sweat. The sensor demonstrated high operational stability over 20 runs of measurements and long-term storage stability over 30 days of storage. Furthermore, the sensor is highly flexible and can withstand 200 cycles of bending without significant changes in its performance. The flexibility of the textile substrate and the ability of the sensor to withstand bending stress enable its integration into clothing for long-term and continuous monitoring of glucose levels.

This textile-based biosensor can serve as a multiplexed sensing platform for personalized healthcare and telemedicine. Given the capabilities of textile-based wearable sensors to provide wearing comfort and withstand mechanical deformation, multiple sensing modalities could be integrated to fit versatile applications, including diabetes management, mental health analysis, fitness monitoring, and general wellness tracking.

Moreover, future work could also focus on the integration of physical, physiological, and electrochemical sensors into this single textile patch for simultaneous, continuous, real-time, long-term monitoring of vital signs such as heart rate, blood pressure, and respiration. Smart textiles can provide a comprehensive view of an individual’s health status. In addition, a wearable Potentiostat will be printed on a flexible printed circuit board (PCB) that can be integrated into fabrics or worn as a wristband. Conductive interconnections (wiring) will be screen printed on the textile, and biosensors will be connected through silver yarns within the textile platform. Low-energy Bluetooth (BLE) will be employed here as a wireless communication tool that can transmit data to a smart phone or upload it to the cloud for further processing. Overall, we will develop a fully integrated platform and test the on-body functionality in the future, ensuring its practicability and reliability. In addition, washability is another critical feature of the textile-based biosensing platform. At this proof-of-concept stage, washability is beyond the scope of this study. However, more investigations will be conducted to seek optimal encapsulation of the sensor for the excellent washability of this platform.

In conclusion, such textile electronics technology has the potential to revolutionize digital healthcare, empower patient outcomes, and enhance quality of life. This wearable textile glucose sensor holds great potential to serve as a platform for a variety of biosensing applications, paving the way for the development of next-generation wearable healthcare devices.

## Data Availability

Data is available upon request.

## Supplementary Materials

The following supporting information can be downloaded at: https://www.mdpi.com/article/10.3390/bios13070684/s1, Figure S1: Amperometry response of the textile-based sensor to bio analytes present in sweat, Figure S2: On body conformality of textile based wearable sensor.

## References

[1] Zhu Y., Hartel M.C., Yu N., Garrido P.R., Kim S., Lee J., Bandaru P., Guan S., Lin H., Emaminejad S. (2021). Epidermis-Inspired Wearable Piezoresistive Pressure Sensors Using Reduced Graphene Oxide Self-Wrapped Copper Nanowire Networks. Small Methods.

[2] Gao X., Chen X., Hu H., Wang X., Yue W., Mu J., Lou Z., Zhang R., Shi K., Chen X. (2022). A photoacoustic patch for three-dimensional imaging of hemoglobin and core temperature. Nat. Commun..

[3] Zhu Y., Haghniaz R., Hartel M.C., Guan S., Bahari J., Li Z., Baidya A., Cao K., Gao X., Li J. (2023). A Breathable, Passive-Cooling, Non-Inflammatory, and Biodegradable Aerogel Electronics for Wearable Physical-Electrophysiological-Chemical Analysis. Adv. Mater..

[4] Luo Y., Abidian M.R., Ahn J.-H., Akinwande D., Andrews A.M., Antonietti M., Bao Z., Berggren M., Berkey C.A., Bettinger C.J. (2023). Technology Roadmap for Flexible Sensors. ACS Nano.

[5] Goud K.Y., Sandhu S.S., Teymourian H., Yin L., Tostado N., Raushel F.M., Harvey S.P., Moores L.C., Wang J. (2021). Textile-based wearable solid-contact flexible fluoride sensor: Toward biodetection of G-type nerve agents. Biosens. Bioelectron..

[6] Kim J., Campbell A.S., de Ávila B.E.-F., Wang J. (2019). Wearable biosensors for healthcare monitoring. Nat. Biotechnol..

[7] International Diabetes Federation (2015). IDF Diabetes Atlas.

[8] Makaram P., Owens D., Aceros J. (2014). Trends in Nanomaterial-Based Non-Invasive Diabetes Sensing Technologies. Diagnostics.

[9] Coster S., Gulliford M.C., Seed P.T., Powrie J.K., Swaminathan R. (2000). Monitoring blood glucose control in diabetes mellitus: A systematic review. Health Technol. Assess..

[10] Chen C., Xie Q., Yang D., Xiao H., Fu Y., Tan Y., Yao S. (2012). Recent advances in electrochemical glucose biosensors: A review. RSC Adv..

[11] Lee H., Hong Y.J., Baik S., Hyeon T., Kim D.-H. (2018). Enzyme-Based Glucose Sensor: From Invasive to Wearable Device. Adv. Healthc. Mater..

[12] Kim J., Campbell A.S., Wang J. (2018). Wearable non-invasive epidermal glucose sensors: A review. Talanta.

[13] Kim J., Sempionatto J.R., Imani S., Hartel M.C., Barfidokht A., Tang G., Campbell A.S., Mercier P.P., Wang J. (2018). Simultaneous Monitoring of Sweat and Interstitial Fluid Using a Single Wearable Biosensor Platform. Adv. Sci..

[14] Yokus M.A., Songkakul T., Pozdin V.A., Bozkurt A., Daniele M.A. (2020). Wearable multiplexed biosensor system toward continuous monitoring of metabolites. Biosens. Bioelectron..

[15] Abellán-Llobregat A., Jeerapan I., Bandodkar A., Vidal L., Canals A., Wang J., Morallón E. (2017). A stretchable and screen-printed electrochemical sensor for glucose determination in human perspiration. Biosens. Bioelectron..

[16] Zhu Y., Li J., Kim J., Li S., Zhao Y., Bahari J., Eliahoo P., Li G., Kawakita S., Haghniaz R. (2023). Skin-interfaced electronics: A promising and intelligent paradigm for personalized healthcare. Biomaterials.

[17] Moyer J., Wilson D., Finkelshtein I., Wong B., Potts R. (2012). Correlation Between Sweat Glucose and Blood Glucose in Subjects with Diabetes. Diabetes Technol. Ther..

[18] Bandodkar A.J., Jia W., Yardımcı C., Wang X., Ramirez J., Wang J. (2015). Tattoo-Based Noninvasive Glucose Monitoring: A Proof-of-Concept Study. Anal. Chem..

[19] Zhu Y., Haghniaz R., Hartel M.C., Mou L., Tian X., Garrido P.R., Wu Z., Hao T., Guan S., Ahadian S. (2021). Recent Advances in Bioinspired Hydrogels: Materials, Devices, and Biosignal Computing. ACS Biomater. Sci. Eng..

[20] De Pascali C., Francioso L., Giampetruzzi L., Rescio G., Signore M.A., Leone A., Siciliano P. (2021). Modeling, Fabrication and Integration of Wearable Smart Sensors in a Monitoring Platform for Diabetic Patients. Sensors.

[21] Sempionatto J.R., Moon J.-M., Wang J. (2021). Touch-Based Fingertip Blood-Free Reliable Glucose Monitoring: Personalized Data Processing for Predicting Blood Glucose Concentrations. ACS Sensors.

[22] Liu H., Zhang S., Li Z., Lu T.J., Lin H., Zhu Y., Ahadian S., Emaminejad S., Dokmeci M.R., Xu F. (2021). Harnessing the wide-range strain sensitivity of bilayered PEDOT:PSS films for wearable health monitoring. Matter.

[23] Malon R.S.P., Chua K.Y., Wicaksono D.H.B., Córcoles E.P. (2014). Cotton fabric-based electrochemical device for lactate measurement in saliva. Analyst.

[24] Zafar H., Channa A., Jeoti V., Stojanović G.M. (2022). Comprehensive Review on Wearable Sweat-Glucose Sensors for Continuous Glucose Monitoring. Sensors.

[25] Chen X., Gao X., Nomoto A., Shi K., Lin M., Hu H., Gu Y., Zhu Y., Wu Z., Chen X. (2021). Fabric-substrated capacitive biopotential sensors enhanced by dielectric nanoparticles. Nano Res..

[26] Luo C., Gil I., Fernandez-Garcia R. (2021). Textile UHF-RFID antenna sensor for measurements of sucrose solutions in different levels of concentration. Meas. Sci. Technol..

[27] Park H., Kim J.W., Hong S.Y., Lee G., Lee H., Song C., Keum K., Jeong Y.R., Jin S.W., Kim D.S. (2019). Dynamically Stretchable Supercapacitor for Powering an Integrated Biosensor in an All-in-One Textile System. ACS Nano.

[28] Sinha A., Dhanjai, Stavrakis A.K., Stojanović G.M. (2022). Textile-based electrochemical sensors and their applications. Talanta.

[29] Piper A., Månsson I., Khaliliazar S., Landin R., Hamedi M.M. (2021). A disposable, wearable, flexible, stitched textile electrochemical biosensing platform. Biosens. Bioelectron..

[30] Liu X., Lillehoj P.B. (2016). Embroidered electrochemical sensors for biomolecular detection. Lab A Chip.

[31] Zhao Y., Zhai Q., Dong D., An T., Gong S., Shi Q., Cheng W. (2019). Highly Stretchable and Strain-Insensitive Fiber-Based Wearable Electrochemical Biosensor to Monitor Glucose in the Sweat. Anal. Chem..

[32] Wang R., Zhai Q., An T., Gong S., Cheng W. (2020). Stretchable gold fiber-based wearable textile electrochemical biosensor for lactate monitoring in sweat. Talanta.

[33] Liu X., Lillehoj P.B. (2017). Embroidered electrochemical sensors on gauze for rapid quantification of wound biomarkers. Biosens. Bioelectron..

[34] Mo L., Guo Z., Yang L., Zhang Q., Fang Y., Xin Z., Chen Z., Hu K., Han L., Li L. (2019). Silver Nanoparticles Based Ink with Moderate Sintering in Flexible and Printed Electronics. Int. J. Mol. Sci..

[35] Bacalzo N.P., Go L.P., Querebillo C.J., Hildebrandt P., Limpoco F.T., Enriquez E.P. (2018). Controlled microwave-hydrolyzed starch as a stabilizer for green formulation of aqueous gold nanoparticle ink for flexible printed electronics. ACS Appl. Nano Mater..

[36] Patil S.A., Ryu C.-H., Kim H.-S. (2018). Synthesis and Characterization of Copper Nanoparticles (Cu-Nps) using Rongalite as Reducing Agent and Photonic Sintering of Cu-Nps Ink for Printed Electronics. Int. J. Precis. Eng. Manuf. Technol..

[37] Ferri J., Llopis R.L., Moreno J., Lidón-Roger J.V., Garcia-Breijo E. (2020). An investigation into the fabrication parameters of screen-printed capacitive sensors on e-textiles. Text. Res. J..

[38] Gong X., Huang K., Wu Y.-H., Zhang X.-S. (2022). Recent progress on screen-printed flexible sensors for human health monitoring. Sens. Actuators A Phys..

[39] Choi J., Bandodkar A.J., Reeder J.T., Ray T.R., Turnquist A., Kim S.B., Nyberg N., Hourlier-Fargette A., Model J.B., Aranyosi A.J. (2019). Soft, Skin-Integrated Multifunctional Microfluidic Systems for Accurate Colorimetric Analysis of Sweat Biomarkers and Temperature. ACS Sens..

[40] Yin L., Cao M., Kim K.N., Lin M., Moon J.-M., Sempionatto J.R., Yu J., Liu R., Wicker C., Trifonov A. (2022). A stretchable epidermal sweat sensing platform with an integrated printed battery and electrochromic display. Nat. Electron..

[41] Kim S., Lee B., Reeder J.T., Seo S.H., Lee S.-U., Hourlier-Fargette A., Shin J., Sekine Y., Jeong H., Oh Y.S. (2020). Soft, skin-interfaced microfluidic systems with integrated immunoassays, fluorometric sensors, and impedance measurement capabilities. Proc. Natl. Acad. Sci. USA.

[42] Pedro B.G., Marcôndes D.W.C., Bertemes-Filho P. (2020). Analytical Model for Blood Glucose Detection Using Electrical Impedance Spectroscopy. Sensors.

[43] Kim Y.-J., Chinnadayyala S.R., Le H.T.N., Cho S. (2022). Sensitive Electrochemical Non-Enzymatic Detection of Glucose Based on Wireless Data Transmission. Sensors.

[44] Xu J., Yan Z., Liu Q. (2022). Smartphone-Based Electrochemical Systems for Glucose Monitoring in Biofluids: A Review. Sensors.

[45] Hassan M.H., Vyas C., Grieve B., Bartolo P. (2021). Recent Advances in Enzymatic and Non-Enzymatic Electrochemical Glucose Sensing. Sensors.

[46] Kim J., Jeerapan I., Sempionatto J.R., Barfidokht A., Mishra R.K., Campbell A.S., Hubble L.J., Wang J. (2018). Wearable Bioelectronics: Enzyme-Based Body-Worn Electronic Devices. Accounts Chem. Res..

[47] Khor S.M., Choi J., Won P., Ko S.H. (2022). Challenges and Strategies in Developing an Enzymatic Wearable Sweat Glucose Biosensor as a Practical Point-Of-Care Monitoring Tool for Type II Diabetes. Nanomaterials.

[48] Katseli V., Economou A., Kokkinos C. (2021). Smartphone-Addressable 3D-Printed Electrochemical Ring for Nonenzymatic Self-Monitoring of Glucose in Human Sweat. Anal. Chem..

[49] Karyakin A.A. (2017). Advances of Prussian blue and its analogues in (bio)sensors. Curr. Opin. Electrochem..

[50] Lu S.-Y., Chen Y., Fang X., Feng X. (2017). Hydrogen peroxide sensor based on electrodeposited Prussian blue film. J. Appl. Electrochem..

[51] Ahmadalinezhad A., Kafi A., Chen A. (2009). Glucose biosensing based on the highly efficient immobilization of glucose oxidase on a Prussian blue modified nanostructured Au surface. Electrochem. Commun..

[52] Cinti S., Arduini F., Moscone D., Palleschi G., Killard A.J. (2014). Development of a Hydrogen Peroxide Sensor Based on Screen-Printed Electrodes Modified with Inkjet-Printed Prussian Blue Nanoparticles. Sensors.

[53] Aller-Pellitero M., Fremeau J., Villa R., Guirado G., Lakard B., Hihn J.-Y., del Campo F.J. (2019). Electrochromic biosensors based on screen-printed Prussian Blue electrodes. Sens. Actuators B Chem..

[54] Lee H., Song C., Hong Y.S., Kim M.S., Cho H.R., Kang T., Shin K., Choi S.H., Hyeon T., Kim D.-H. (2017). Wearable/disposable sweat-based glucose monitoring device with multistage transdermal drug delivery module. Sci. Adv..

[55] De la Paz E., Barfidokht A., Rios S., Brown C., Chao E., Wang J. (2021). Extended noninvasive glucose monitoring in the interstitial fluid using an epidermal biosensing patch. Anal. Chem..

[56] Wiorek A., Parrilla M., Cuartero M., Crespo G.A. (2020). Epidermal Patch with Glucose Biosensor: pH and Temperature Correction toward More Accurate Sweat Analysis during Sport Practice. Anal. Chem..

[57] Yin J., Li J., Reddy V.S., Ji D., Ramakrishna S., Xu L. (2023). Flexible Textile-Based Sweat Sensors for Wearable Applications. Biosensors.

[58] Lee H., Choi T.K., Lee Y.B., Cho H.R., Ghaffari R., Wang L., Choi H.J., Chung T.D., Lu N., Hyeon T. (2016). A graphene-based electrochemical device with thermoresponsive microneedles for diabetes monitoring and therapy. Nat. Nanotechnol..

[59] Emaminejad S., Gao W., Wu E., Davies Z.A., Nyein H.Y.Y., Challa S., Ryan S.P., Fahad H.M., Chen K., Shahpar Z. (2017). Autonomous sweat extraction and analysis applied to cystic fibrosis and glucose monitoring using a fully integrated wearable platform. Proc. Natl. Acad. Sci. USA.

[60] Nyein H.Y.Y., Tai L.-C., Ngo Q.P., Chao M., Zhang G.B., Gao W., Bariya M., Bullock J., Kim H., Fahad H.M. (2018). A Wearable Microfluidic Sensing Patch for Dynamic Sweat Secretion Analysis. ACS Sens..

[61] Zhu Y., Nasiri R., Davoodi E., Zhang S., Saha S., Linn M., Jiang L., Haghniaz R., Hartel M.C., Jucaud V. (2022). A Microfluidic Contact Lens to Address Contact Lens-Induced Dry Eye. Small.

[62] Li S., Zhu Y., Haghniaz R., Kawakita S., Guan S., Chen J., Li Z., Mandal K., Bahari J., Shah S. (2022). A Microchambers Containing Contact Lens for the Noninvasive Detection of Tear Exosomes. Adv. Funct. Mater..

[63] Koh A., Kang D., Xue Y., Lee S., Pielak R.M., Kim J., Hwang T., Min S., Banks A., Bastien P. (2016). A soft, wearable microfluidic device for the capture, storage, and colorimetric sensing of sweat. Sci. Transl. Med..

[64] Shajari S., Salahandish R., Zare A., Hassani M., Moossavi S., Munro E., Rashid R., Rosenegger D., Bains J.S., Nezhad A.S. (2023). MicroSweat: A Wearable Microfluidic Patch for Noninvasive and Reliable Sweat Collection Enables Human Stress Monitoring. Adv. Sci..

[65] Wu C.-H., Ma H.J.H., Baessler P., Balanay R.K., Ray T.R. (2023). Skin-interfaced microfluidic systems with spatially engineered 3D fluidics for sweat capture and analysis. Sci. Adv..

[66] Boubin M., Shrestha S. (2019). Microcontroller Implementation of Support Vector Machine for Detecting Blood Glucose Levels Using Breath Volatile Organic Compounds. Sensors.

[67] Han L., Luo S., Yu J., Pan L., Chen S. (2014). Rule Extraction from Support Vector Machines Using Ensemble Learning Approach: An Application for Diagnosis of Diabetes. IEEE J. Biomed. Health Inform..

[68] Karan O., Bayraktar C., Gümüşkaya H., Karlık B. (2012). Diagnosing diabetes using neural networks on small mobile devices. Expert Syst. Appl..

[69] Temurtas H., Yumusak N., Temurtas F. (2009). A comparative study on diabetes disease diagnosis using neural networks. Expert Syst. Appl..

[70] Sarría-Santamera A., Orazumbekova B., Maulenkul T., Gaipov A., Atageldiyeva K. (2020). The Identification of Diabetes Mellitus Subtypes Applying Cluster Analysis Techniques: A Systematic Review. Int. J. Environ. Res. Public Health.

[71] Alamsyah M., Nafisah Z., Prayitno E., Afida A.M., Imah E.M. (2018). The Classification of Diabetes Mellitus Using Kernel k-means. J. Phys. Conf. Ser..

