# Supplementary material for: Screen-Printed Textile-Based Electrochemical Biosensor for Noninvasive Monitoring of Glucose in Sweat
Source: Biosensors (Basel). 2023 Jun 27;13(7):684. doi: 10.3390/bios13070684 (PMC10377550; doi:10.3390/bios13070684)
Supplement: Supplementary file 1 [file biosensors-13-00684-s001.zip › biosensors-2391197-supplementary.pdf]

biosensors

MDPI

Supplementary Material

Screen-Printed Textile-Based Electrochemical Biosensor for Noninvasive Monitoring of Glucose in Sweat

Safoora Khosravi, Saeid Soltanian, Amir Servati, Ali Khademhosseini, Yangzhi Zhu, Peyman Servati

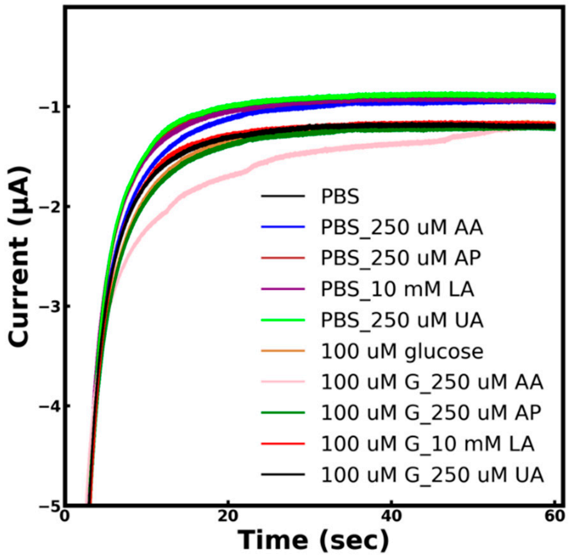

Figure S1. Amperometry response of the textile-based sensor to bio analytes present in sweat.

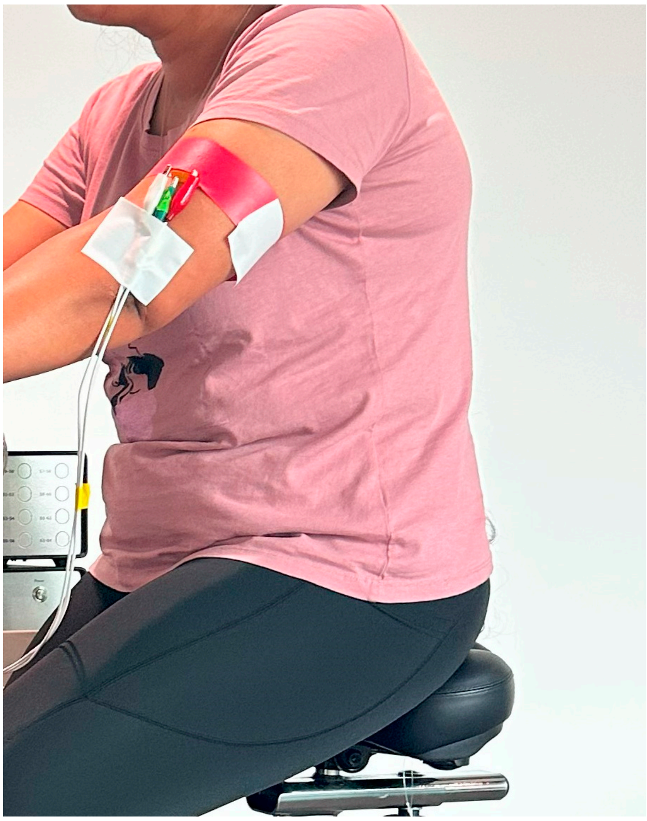

Figure S2. On body conformality of textile based wearable sensor.
